# The effect of caregiver mastery on the associations of depression, anxiety, caregiver burden, fear of disease progression with quality of life among children with solid tumors

**DOI:** 10.1017/S1478951524001998

**Published:** 2025-01-21

**Authors:** Fangfang Cheng, Rui Gao, Huanhuan Zhu, Yu Zhang, Hui Xiong, Yingtao Meng

**Affiliations:** 1 Department of pediatrics, Shandong Cancer Hospital and Institute, Shandong First Medical University and Shandong Academy of Medical Sciences, Jinan, Shandong, P.R. China; 2 Nursing Department, Taian Disabled Soldiers’ The Second Veterans Special Care Hospital of Shandong Province, Taian, Shandong, P.R. China; 3 Nursing Department, Shandong Cancer Hospital and Institute, Shandong First Medical University and Shandong Academy of Medical Sciences, Jinan, Shandong, P.R. China

**Keywords:** Caregiver mastery, quality of life, solid tumor, children, family caregivers

## Abstract

**Objectives:**

Caring for children with solid tumors (STs) can impact caregiver’s physical and mental health. Caregiver mastery, which influences psychological well-being, is vital in improving outcomes for both caregivers and children. The study aimed to investigate the relationship between caregiver mastery, anxiety, depression, fear of disease progression (FoP), caregiver burden, and the quality of life (QOL) of children with ST.

**Methods:**

This cross-sectional study was conducted from June 2022 to April 2023 at a Grade A tertiary hospital in Shandong. Family caregivers of children with ST completed several validated measures, including the Pediatric Quality of Life Inventory (PedsQL) 3.0 Cancer Module, the Fear of Progression Questionnaire-parent version (FoP-Q-SF/PR), the Zarit Burden Interview Scale (ZBI), the hospital anxiety and depression scale (HADS), and the Caregiver Mastery Scale. Multiple linear regression analyses assessed the relationships between FoP, caregiver burden, anxiety, depression, caregiver mastery, and children’s QOL. Results were expressed as *β* and 95% confidence intervals (CIs).

**Results:**

A total of 454 caregivers participated. Caregiver mastery was positively correlated with children’s QOL (*β* = 0.80, 95% CI: 0.20 to 1.39). Depression (*β* = −0.64, 95% CI: −0.83 to −0.45), anxiety (*β* = −0.67, 95% CI: −0.85 to −0.49), caregiver burden (*β* = −1.20, 95% CI: −1.60 to −0.80), and FoP (*β* = −0.04, 95% CI: −0.05 to −0.03) were negatively related to children’s QOL. Caregiver mastery moderated the associations between depression, caregiver burden, FoP, and children’s QOL, while also improving the effect of mild anxiety on QOL.

**Significance of results:**

The study underscores the importance of fostering caregiver mastery to mitigate the negative impact of caregiver distress on children’s QOL and improve outcomes for both caregivers and children with solid tumors.

**Conclusion:**

Caregiver mastery moderates the effects of anxiety, depression, FoP, and caregiver burdenon children’s QOL. Supporting caregiver mastery can alleviate caregiver burden and enhance both caregiver and child well-being.

## Introduction

Childhood malignant tumors are life-threatening conditions, currently the second leading cause of death in children after accidental trauma (Cunningham et al. 2018). Solid tumors (STs) account for nearly 60% of all pediatric malignancies, emerging as abnormal cellular growth in local tissues due to various carcinogenic factors and genetic aberrations, often presenting as space-occupying masses (Melaiu et al. 2022; Nyagetuba and Hansen 2017). Children with malignant tumors face significant physical, psychological, social, and quality of life (QOL) challenges compared to their healthy peers. QOL for these children encompasses their overall well-being, including participation in shared activities, social interactions, feeling cared for, coping with distressing physical and emotional symptoms, and finding meaning in their illness experience (Hinds et al. 2004). The definition of QOL in children with ST considers emotional adaptation to the experience of malignancy, symptom effects, and the influence of caregivers and parents as primary factors (Hinds et al. 2004).

Caregiver mastery reflects the caregiver’s self-perception of their effectiveness in caregiving or confidence (Lawton et al. 1989). Caregiver mastery influences the caregiver’s coping strategies, adjustments, and adaptions to various situations (Boele et al. 2017; Loh et al. 2021). Inadequate caregiver mastery has been linked to psychological distress (Kabia et al. 2022). Previous studies indicate that higher levels of mastery among caregivers of patients with malignancies were correlated with improved health outcomes, reduced anxiety and depression, decreased physiological stress response, and lower caregiver burden (Chan et al. 2018; Cox et al. 2018; Loh et al. 2021). Improved caregiver mastery not only enhances the caregiver’s well-being but also influences patients’ well-being for the better (Boele et al. 2017).

Fear of progression (FoP) is acknowledged as a reactive and conscious concern arising from chronic illnesses such as cancer (Dinkel and Herschbach 2018). FoP is a prevalent long-term psychological issue among parents of cancer patients (Ljungman et al. 2014). FoP intensifies psychological distress in caregivers, influencing their physical and mental well-being, and reducing the quality of care provided, thus ultimately impacting the patient’s QOL (De Schepper et al. 2016; Mellon et al. 2007).

Our study aimed to explore the relationships between depression, anxiety, FoP, caregiver burden, caregiver mastery, and QOL in children with ST. Additionally, we aimed to investigate the moderating effect of caregiver mastery on the associations of depression, anxiety, FoP, and caregiver burden, with QOL in children. The findings would provide a theoretical foundation for enhancing caregiver mastery in parents of children with ST.

## Methods

### Study design, setting, and participants

This cross-sectional study involved 454 family caregivers of children with ST, selected through convenience sampling between October 2022 and June 2023. Participants were recruited from a Grade A tertiary hospital in Jinan, Shandong Province, China. Inclusion was based on the following: (1) the children aged 2–18 years, (2) the diagnosis of children with ST meets international classification (Steliarova-Foucher et al. 2005), (3) caregivers informed consent and cooperation in completing the questionnaire. The exclusion criteria included children with other diseases and individuals with writing or hearing impairments who could not complete the questionnaire.

### Measurement tools

Dependent variable: The Pediatric Quality of Life Inventory (PedsQL) 3.0 Cancer Module was designed to evaluate the specific impact of cancer on the QOL among children aged 2–18 years, including both children and parent reports (Scarpelli et al. 2008). The PedsQL 3.0 Cancer Module has been categorized into 2 parallel versions for different age groups: child reports (5–7, 8–12, and 13–18 years) and parent reports (2–4, 5–7, 8–12, and 13–18 years). The PedsQL 3.0 Cancer Module consists of 27 items in 8 domains covered by 5 scales: pain and hurt (2 items), nausea (5 items), procedural anxiety (3 items), treatment anxiety (3 items), worry (3 items), cognitive problems (5 items), perceived physical appearance (3 items), and communication (3 items). In the version of PedsQL reported by children aged 5–7 years old, a 3-point Likert scale (0 = never, 2 = sometimes, and 4 = almost always) combined with visual representations (happy, neutral, and sad faces) was employed. For other versions of PedsQL, a 5-point Likert scale (ranging from 0 = never to 4 = almost always) was used. The score for each item was reversed and linearly transformed to a 0–100 scale (0 = 100, 1 = 75, 3 = 25, 4 = 0), with higher scores indicating better QOL. The Cronbach’s *α* was 0.762 in this study.

Independent variable: Fear of Progression Questionnaire-parent version (FoP-Q-SF/PR) was an adaption of Fear of Progression Questionnaire (FoP-Q-SF) for the parents of children with cancer (Schepper et al. 2015). The scale, consisting of 12 items, was rated on a 5-point scale from “never” to “always.” Total scores of FoP were calculated by aggregating scores for each item, ranging from 12 to 60, with a higher score indicating a higher level of FoP. The Cronbach’s *α* was 0.895 in the Chinese version of the FoP-Q-SF/PR (Yang et al. 2022).

The Zarit Burden Interview Scale (ZBI) was a 22-item scale designed for caregivers to assess the burden of caring for their children, with responses ranging from “never” (0) to “always” (4) (Zarit et al. 1986). The total score ranged from 0 to 88 with higher scores indicating a high level of caregiver burden. The severity of the burden was classified into 3 levels of the total ZBI scores: “little or no burden” (0–40), “moderate” (41–60), and “severe” (>60). The Chinese version of ZBI has excellent psychometric properties (Li et al. 2018).

Caregiver anxiety and depression were measured using the hospital anxiety and depression scale (HADS) consisting of 14 items (Bjelland et al. 2002). The scale consists of two 7-item subscales to evaluate anxiety and depression. Each item was scored on a 4-point scale, total scores ranged from 0 to 21. Subscale total scores above 8 defined anxiety or depression with a higher score indicating a high level of symptoms. The Cronbach’s *α* was 0.84 in the Chinese version (Li et al. 2016).

Moderator: Caregiver mastery was measured using the Chinese Version of the Caregiver Mastery Scale (Cox et al. 2018). This is a 7-item self-reported instrument, and each item was assessed on a 5-point scale from strongly disagree (1) to strongly agree (5). The total score ranged from 7 to 35, with higher scores indicating higher levels of caregiver mastery. Three items with negative statements are reverse-scored. The Cronbach’s *α* for the scale value was 0.92 (Ding et al. 2022).

Covariates: Covariates included demographics of the child and caregiver, as well as disease-related information about the child. Caregiver demographics were age, relationship to the child, residence, marital status, education, monthly household income, working status, health status, number of family members over 60 years, and children <18 years old in the household. The child demographics were gender and age. Disease-related information about the child included the time of diagnosis, the time of initiation of treatment, the type of treatment, the duration of treatment, and the status of tumor recurrence or metastasis.

### Statistical analysis

Categorical variables were presented as numbers and percentages. Skewness and kurtosis were used to examine the normality of quantitative data, and Levene tests were conducted to test the equality of variances. Continuous variables were expressed as means and standard deviation for normality, median, and quartile for abnormality. Potential covariates were screened using univariate linear regression analysis. A rank sum test was used to compare the differences in scores of QOL between parents and children. Multivariate linear regression analyses were conducted to investigate the association between independent (FoP, caregiver burden, anxiety, and depression), moderator (caregiver mastery), and dependent (QOL of children) variables. All analyses were performed using SAS 9.4 (SAS Institute Inc., Cary, NC, USA), and *P* < 0.05 was considered statistically significant.

## Results

### Characteristics of caregivers for children with STs

Table 1 shows the characteristics of participants. The median age of children with ST was 6 years, with 263 (57.93%) males. The median duration of treatment was 10 months. Among those children, 302 (66.52%) had developed tumor metastasis, and 329 (72.47%) had suffered surgeries. The caregivers of children with ST were predominantly mothers (*n* = 371, 81.72%), with a mean age of 36.83 (±10.80) years. Among caregivers, 235 (51.76%) lived in rural, 425 (93.61%) were married, 207 (45.59%) had attained a junior degree or above. Among caregivers, only 121 (26.65%) have full-time jobs, and 200 (44.05%) have a monthly income of <1000 Yuan. Of children diagnosed with ST, 254 (55.95%) were the firstborn in the families. And most families have 2 or more children.

**Table 1.** Characteristics of family caregivers for children with solid tumors

Variables	Total (*n* = 454)
Age, *M* (*Q*_1_, *Q*_3_)	6.00 (4.00, 10.00)
Age, years, *n* (%)	
2–4	139 (30.62)
5–7	131 (28.85)
8–12	117 (25.77)
13–18	67 (14.76)
Gender, *n* (%)	
Male	263 (57.93)
Female	191 (42.07)
Duration of treatment, month, *M* (*Q*_1_, *Q*_3_)	10.00 (5.00, 20.00)
Relationship to the child, *n* (%)	
Father	73 (16.08)
Mother	371 (81.72)
Grandpa/Grandma/Sister	10 (2.20)
Age of mother, Mean ± SD	36.83 ± 6.23
Residence, *n* (%)	
Urban	219 (48.24)
Rural	235 (51.76)
Marital status, *n* (%)	
Others	29 (6.39)
Married	425 (93.61)
Education, *n* (%)	
Junior high school and below	146 (32.16)
High school/technical secondary school	101 (22.25)
Junior college/Regular college and above	207 (45.59)
Monthly household income (RMB), *n* (%)	
<1000	200 (44.05)
1000–5000	172 (37.89)
5000–10,000	58 (12.78)
>10,000	24 (5.29)
Health status, *n* (%)	
Good	328 (72.25)
General/Poor	126 (27.75)
Family members aged 60 and above, *n* (%)	
0	64 (14.10)
1	70 (15.42)
2	181 (39.87)
3	51 (11.23)
More than 4	88 (19.38)
Children <18 years old in the household, *n* (%)	
1 or less	144 (31.72)
2	272 (59.91)
3 or more	38 (8.37)
Ranking of the child with ST in family, *n* (%)	
First	254 (55.95)
Second	181 (39.87)
Third or more	19 (4.19)
Metastasized, *n* (%)	
No	152 (33.48)
Yes	302 (66.52)
Caregiver burden, *n* (%)	
Little or no burden	274 (60.35)
Moderate	140 (30.84)
Severe	40 (8.81)
Occupation, *n* (%)	
Part-time job	104 (22.91)
Full-time job	121 (26.65)
Unemployed/ retired	229 (50.44)
Combination therapy, *n* (%)	
No	115 (25.33)
Yes	339 (74.67)
Radiation, *n* (%)	
No	236 (51.98)
Yes	218 (48.02)
Surgery, *n* (%)	
No	125 (27.53)
Yes	329 (72.47)
Chemotherapy, *n* (%)	
No	15 (3.30)
Yes	439 (96.70)
Depression, *n* (%)	
No	95 (20.93)
Yes	359 (79.07)
Anxiety, *n* (%)	
No	115 (25.33)
Yes	339 (74.67)
Total score of HADS-anxiety, *M* (*Q*_1_, *Q*_3_)	10.00 (7.00, 14.00)
Total score of HADS-depression, *M* (*Q*_1_, *Q*_3_)	11.00 (8.00, 14.00)
Total score of FoP-Q-SF/PR, Mean ± SD	38.38 ± 10.12
Total score of caregiver burden, *M* (*Q*_1_, *Q*_3_)	35.00 (25.00, 45.00)
Total score of caregiver mastery, Mean ± SD	21.65 ± 2.59

SD: standard deviation, *M*: median, *Q*_1_: 1st quartile, *Q*_3_: 3rd quartile, HADS: hospital anxiety and depression scale, FoP-Q-SF/PR: Fear of Progression Questionnaire-parent version.

### Scores on QOL, anxiety, depression, caregiver mastery, FoP, and caregiver burden

Median QOL scores reported by children and parents were 57.41 (range: 48.15–69.44) and 53.71 (range: 41.67–64.81), respectively, indicating that the QOL level of the children was moderate. The scores for treatment anxiety and worry in the subcategories of PedsQL 3.0 Cancer Module were statistically different between child and parent reports (*P* < 0.001) (Table 2). The average score of caregiver mastery was 21.65 ± 2.59, and the specific scores for 7 items were shown in Table 3. The median scores for anxiety and depression were 10.00 and 11.00, respectively. The mean FoP score was 38.38 ± 10.12, indicating a slightly high level of FoP. The median score for caregiver burden was 35.00, indicating a moderate level of burden.

**Table 2.** Scores of quality of life reported by children and parents

	Reported			
Variables	Children (*n* = 315)	Parents (*n* = 315)	Statistics	*P*
Total score, *M* (*Q*_1_, *Q*_3_)	57.41 (48.15, 69.44)	53.70 (41.67, 64.81)	*Z* = 3.340	<0.001
Pain and hurt, *M* (*Q*_1_, *Q*_3_)	62.50 (50.00, 75.00)	62.50 (50.00, 75.00)	*Z* = −0.314	0.753
Nausea, *M* (*Q*_1_, *Q*_3_)	50.00 (30.00, 60.00)	50.00 (30.00, 60.00)	*Z* = 0.754	0.451
Procedural anxiety, *M* (*Q*_1_, *Q*_3_)	50.00 (25.00, 66.67)	50.00 (25.00, 66.67)	*Z* = −0.285	0.776
Treatment anxiety, *M* (*Q*_1_, *Q*_3_)	58.33 (50.00, 83.33)	50.00 (33.33, 75.00)	*Z* = 4.094	<0.001
Worry, *M* (*Q*_1_, *Q*_3_)	50.00 (33.33, 83.33)	41.67 (16.67, 58.33)	*Z* = 6.499	<0.001
Cognitive problems, *M* (*Q*_1_, *Q*_3_)	65.00 (50.00, 85.00)	60.00 (50.00, 75.00)	*Z* = 1.354	0.176
Perceived physical appearance, *M* (*Q*_1_, *Q*_3_)	58.33 (41.67, 83.33)	58.33 (41.67, 75.00)	*Z* = 1.540	0.124
Communication, *M* (*Q*_1_, *Q*_3_)	66.67 (50.00, 100.00)	58.33 (50.00, 75.00)	*Z* = 1.861	0.063

*Z*: rank sum test, *M*: median, *Q*_1_: 1st quartile, *Q*_3_: 3rd quartile.

**Table 3.** The score of caregiver mastery

Variables	Strongly disagree	Disagree	Hard judge	Agree	Strongly agree
You are usually certain about what to do in caring for your child(ren), *n* (%)	3 (0.66)	6 (1.32)	59 (13.00)	278 (61.23)	108 (23.79)
No matter what you do as a caregiver, it never seems to be enough, *n* (%)	123 (27.09)	239 (52.64)	48 (10.57)	40 (8.81)	4 (0.88)
In general, you are able to handle most problems in the care of your child(ren), *n* (%)	0 (0.00)	20 (4.41)	82 (18.06)	298 (65.64)	54 (11.89)
You are not doing as well as you would like as a caregiver, *n* (%)	57 (12.56)	229 (50.44)	95 (20.93)	68 (14.98)	5 (1.10)
You feel that you have a great deal of influence over the things that happen in caregiving, *n* (%)	5 (1.10)	46 (10.13)	139 (30.62)	218 (48.02)	46 (10.13)
You believe you are mastering most of the challenges in caregiving, *n* (%)	4 (0.88)	37 (8.15)	150 (33.04)	225 (49.56)	38 (8.37)
You have lost some control of your life since your child(ren) was sick, *n* (%)	100 (22.03)	246 (54.19)	48 (10.57)	53 (11.67)	7 (1.54)

### Relationship between caregiver mastery, depression, anxiety, caregiver burden, FoP, and QOL of children

Caregiver mastery was positively correlated with the QOL of children (*β* = 0.80, 95% CI: 0.20 to 1.39) (Table 4). After adjusting for covariates, caregiver mastery was positively correlated with procedural anxiety (*β* = 1.85, 95% CI: 0.88 to 2.82), treatment anxiety (*β* = 1.42, 95% CI: 0.39 to 2.46), perceived physical appearance (*β* = 1.06, 95% CI: 0.15 to 1.97), and communication (*β* = 1.49, 95% CI: 0.48 to 2.51) (Table 5). After adjusted for residence, education, income, state of health, and ranking of the child with ST in family, depression (*β* = −0.64, 95% CI: −0.83 to −0.45), anxiety (*β* = −0.67, 95% CI: −0.85 to −0.49), caregiver burden (*β* = −1.20, 95% CI: −1.60 to −0.80), and FoP (*β* = −0.04, 95% CI: −0.05 to −0.03) were negatively related to the QOL of children (Table 6).

**Table 4.** Association between caregiver mastery and quality of life of children with solid tumors

Model	*β* (95% CI)	*P*
Model 1	1.13 (0.59–1.67)	<0.001
Model 2	0.80 (0.20–1.39)	0.009

Model 1: crude model. Model 2: adjusting residence, education, income, state of health, and the child was the how manieth child in family.

**Table 5.** Association between caregiver mastery and 8 domains of quality of life

	Model 1		Model 2	
Group	*β* (95% CI)	*P*	*β* (95% CI)	*P*
Pain and hurt	0.41 (−0.45 to 1.28)	0.350	0.45 (−0.45 to 1.35)	0.329
Nausea	0.56 (−0.24 to 1.36)	0.174	0.73 (−0.07 to 1.54)	0.075
Procedural anxiety	2.17 (1.14 to 3.19)	<0.001	1.85 (0.88 to 2.82)	<0.001
Treatment anxiety	1.59 (0.60 to 2.59)	0.002	1.42 (0.39 to 2.46)	0.007
Worry	0.80 (−0.33 to 1.94)	0.165	0.28 (−0.90 to 1.45)	0.644
Cognitive problems	0.61 (−0.26 to 1.49)	0.169	0.22 (−0.66 to 1.10)	0.626
Perceived physical appearance	1.52 (0.60 to 2.44)	0.001	1.06 (0.15 to 1.97)	0.023
Communication	1.86 (0.87 to 2.86)	<0.001	1.49 (0.48 to 2.51)	0.004

Adjustment for confounders in 8 domains of quality of life: Pain and hurt: duration of treatment, state of health, number of children, the child was the how manieth child in family, metastasized, radiation, and chemotherapy. Nausea: duration of treatment, age, income, state of health, metastasized, and radiation. Procedural anxiety: age, age of mother, and radiation. Treatment anxiety: age and education. Worry: residence, education, and income. Cognitive problems: age, gender, age of mother, residence, education, number of children, and the child was the how manieth child in family. Perceived physical appearance: age, age of mother, residence, education, income, state of health, and number of children. Communication: residence, income, and the child was the how manieth child in family.

**Table 6.** Association between anxiety, depression, caregiver burden, FoP, caregiver mastery, and QOL of children

	Model 1		Model 2	
Variable	*β* (95% CI)	*P*	*β* (95% CI)	*P*
Caregiver mastery	0.07 (0.04 to 0.10)	<0.001	0.05 (0.01 to 0.08)	0.009
Depression				
No	Ref		Ref	
Yes	−0.71 (−0.90 to −0.52)	<0.001	−0.64 (−0.83 to −0.45)	<0.001
Depression				
No	Ref		Ref	
Mild	−0.56 (−0.78 to −0.33)	<0.001	−0.51 (−0.74 to −0.27)	<0.001
Moderate	−0.59 (−0.80 to −0.37)	<0.001	−0.53 (−0.75 to −0.31)	<0.001
Severe	−1.02 (−1.30 to −0.74)	<0.001	−0.95 (−1.23 to −0.67)	<0.001
Anxiety				
No	Ref		Ref	
Yes	−0.70 (−0.88 to −0.52)	<0.001	−0.67 (−0.85 to −0.49)	<0.001
Anxiety				
No	Ref		Ref	
Mild	−0.38 (−0.60 to −0.16)	<0.001	−0.39 (−0.60 to −0.18)	<0.001
Moderate	−0.69 (−0.91 to −0.48)	<0.001	−0.67 (−0.88 to −0.45)	<0.001
Severe	−1.15 (−1.42 to −0.88)	<0.001	−1.10 (−1.38 to −0.82)	<0.001
Caregiver burden				
Little or no burden	Ref		Ref	
Moderate	−0.57 (−0.75 to −0.39)	<0.001	−0.54 (−0.72 to −0.36)	<0.001
Severe	−1.29 (−1.68 to −0.90)	<0.001	−1.20 (−1.60 to −0.80)	<0.001
Total score of FoP	−0.04 (−0.05 to −0.03)	<0.001	−0.04 (−0.05 to −0.03)	<0.001

Ref: reference, CI: confidence interval, FoP: fear of disease progression, QOL: quality of life. Model 1, crude model. Model 2, adjusting residence, education, income, state of health, and the child was the how manieth child in family.

### The moderating role of caregiver mastery

The moderating effects of caregiver mastery are illustrated in Table 7. Covariates were adjusted including education, income, state of health, and ranking of the child with ST in family. When caregivers had high mastery, depression was less likely to impact the QOL of children (*β* = −0.72, 95% CI: −0.99 to −0.44). Similarly, when caregivers had high mastery, anxiety was also less likely to impact the QOL of children (*β* = −0.62, 95% CI: −0.95 to −0.30). And caregiver burden would be less likely to impact the QOL of children (*β* = −1.65, 95% CI: −2.79 to −0.51). In contrast, caregiver mastery didn’t moderate the association between FoP and children’s QOL (*β* = −0.04, 95% CI: −0.06 to −0.03).

**Table 7.** Moderating effect of caregiver mastery on the relationship of anxiety, depression, caregiver burden, FoP, caregiver mastery, with QOL of children

	Low level of caregiver mastery (≤22)		High level of caregiver mastery (>22)	
Variable	*β* (95% CI)	*P*	*β* (95% CI)	*P*
Depression				
No	Ref		Ref	
Yes	−0.56 (−0.83 to −0.29)	<0.001	−0.72 (−0.99 to −0.44)	<0.001
Depression				
No	Ref		Ref	
Mild	−0.48 (−0.78 to −0.17)	0.002	−0.43 (−0.78 to −0.09)	0.016
Moderate	−0.42 (−0.72 to −0.12)	0.007	−0.79 (−1.12 to −0.46)	<0.001
Severe	−0.78 (−1.12 to −0.45)	<0.001	−1.20 (−1.88 to −0.51)	<0.001
Anxiety				
No	Ref		Ref	
Yes	−0.67 (−0.90 to −0.44)	<0.001	−0.62 (−0.95 to −0.30)	<0.001
Anxiety				
No	Ref		Ref	
Mild	−0.45 (−0.72 to −0.18)	0.001	−0.22 (−0.55 to 0.12)	0.206
Moderate	−0.65 (−0.92 to −0.38)	<0.001	−0.64 (−1.04 to −0.24)	0.002
Severe	−0.98 (−1.29 to −0.67)	<0.001	−1.52 (−2.18 to −0.85)	<0.001
Caregiver burden				
Little or no burden	Ref		Ref	
Moderate	−0.41 (−0.62 to −0.20)	<0.001	−0.76 (−1.12 to −0.41)	<0.001
Severe	−1.03 (−1.44 to −0.62)	<0.001	−1.65 (−2.79 to −0.51)	0.005
FoP	−0.04 (−0.05 to −0.03)	<0.001	−0.04 (−0.06 to −0.03)	<0.001

Ref: reference, CI: confidence interval, FoP: fear of disease progression, QOL: quality of life. Adjusting residence, education, income, state of health, and the child was the how manieth child in family.

## Discussion

Our study investigated the impact of family caregiver mastery on the health of children afflicted with ST. We observed a moderate level of QOL among those children. Caregiver mastery has a moderating effect on the associations of anxiety, depression, caregiver burden, with the QOL among children with ST.

Caregiver mastery reflects the caregiver’s self-perception of their efficacy in caregiving or confidence (Lawton et al. 1989). Our study found that caregiver mastery was positively correlated with QOL in children with ST. Specifically, caregiver mastery was significantly influenced by domains in procedural anxiety, treatment anxiety, perceived physical appearance, and communication of QOL in children with ST. Caregivers who possess higher levels of mastery are likely to be more adept at navigating the complexities associated with their children’s illnesses, including medical procedures and treatment-related anxieties (Liu et al. 2023). In addition, caregivers who feel competent in their role are more likely to foster open and effective communication with their children (Hwang et al. 2023). This enhanced communication can help children express their concerns about their physical appearance or any difficulties they may face due to their illness, leading to better psychological adjustment and improved QOL.

Caregiver depression can impact not only the caregiver’s well-being but also that of the children they care for. High levels of depression among caregivers have been consistently linked with poorer QOL among children with ST (Alaqeel et al. 2022). The presence of caregiver mastery appears to mitigate these negative effects. One plausible explanation for this moderating effect lies in the adaptive coping mechanisms that caregiver mastery fosters. Caregivers with higher levels of mastery may exhibit greater resilience in the face of stressors such as depression, thereby maintaining a more stable caregiving environment (Seyedreza et al. 2021). This stability can positively influence the children’s QOL by providing consistent emotional support and practical assistance, which is crucial during the challenging treatment phases associated with ST. Moreover, caregiver mastery might enhance the caregiver’s ability to engage in effective problem-solving and decision-making processes related to the child’s care. This proactive approach can mitigate the detrimental impact of caregiver depression on the child’s emotional and physical well-being (Desjardins et al. 2022). By maintaining a sense of efficacy in their caregiving role, caregivers may also foster a more positive outlook within the family environment, which contributes to improved QOL outcomes for the child (Kan et al. 2021). Additionally, caregiver mastery may promote better communication and collaboration with healthcare providers or children, ensuring that the child receives optimal care tailored to their specific needs and preferences (Loh et al. 2021).

Similarly, caregiver mastery emerged as a moderator in the relationship between anxiety and QOL among children with ST. High levels of anxiety may lead to increased stress, emotional exhaustion, and reduced ability to attend to the children’s needs effectively (Gurtovenko et al. 2021). The negative emotions could inadvertently affect the children’s QOL, as the children may sense the caregiver’s distress (Sayal et al. 2023). Caregivers with high mastery may be more adept at managing their anxiety symptoms, and more proactive in seeking information about the children’s condition, adhering to treatment regimens, and advocating for the children’s needs within the healthcare system (Chan et al. 2018; Greer et al. 2018). Moreover, caregiver mastery could enhance the caregiver–child relationship, fostering open communication and emotional support, which are crucial for maintaining the children’s emotional well-being and QOL (Fenton et al. 2022).

Caregivers of children with ST often face substantial burdens, including managing complex treatment regimens, coping with children’s symptoms, and balancing caregiving responsibilities with other aspects of life (Stieb et al. 2018). High levels of caregiver burden can lead to increased stress, fatigue, and diminished emotional well-being. Caregiver mastery is associated with increased resilience and adaptability in managing stressful situations (Lim et al. 2022; Montpetit and Tiberio 2016; Shin and Park 2024). Caregivers may adapt positively to the challenges imposed by the caregiving burden. The adaptability of caregivers can prevent the burden from overwhelming them, thus preserving their capacity to attend to the child’s physical and emotional needs, which are critical determinants of QOL (Bangerter et al. 2019).

Despite these findings, this study still has several limitations. First, this is a cross-sectional study, which restricts further exploration of the causal relationship between mastery and other variables. Therefore, future investigations should encompass longitudinal studies. Second, the scope of participants was limited from Shandong Province, limiting the generalization of our findings. Large sample and multi-center studies needed to be conducted in the future. Third, our study used self-reported data, which may introduce certain biases in the information provided.

## Conclusion

Caregiver mastery plays a moderating role on the relationship between anxiety/depression/caregiver burden and QOL among children with ST. Our findings suggested the benefits of caregiver mastery in the treatment of children with ST. The study provides a basis for developing a practical caregiver mastery program that can help caregivers increase positive emotions and decrease anxiety, which ultimately improves the QOL of the children.

## References

[ref1] Alaqeel M, Alkhathaami F, Alshangiti A, et al. (2022) Depression and quality of life among caregivers of pediatric cancer patients. *Cureus* 14, e24256. doi:10.7759/cureus.24256. PMC9117091 35602782

[ref2] Bangerter LR, Griffin JM and Dunlay SM (2019) Positive experiences and self-gain among family caregivers of persons with heart failure. *The Gerontologist* 59, e433–e440. doi:10.1093/geront/gny162 30535012 PMC6850888

[ref3] Bjelland I, Dahl AA, Haug TT, et al. (2002) The validity of the hospital anxiety and depression scale. An updated literature review. *Journal of Psychosomatic Research* 52, 69–77. doi:10.1016/s0022-3999(01)00296-3 11832252

[ref4] Boele FW, Given CW, Given BA, et al. (2017) Family caregivers’ level of mastery predicts survival of patients with glioblastoma: A preliminary report. *Cancer* 123, 832–840. doi:10.1002/cncr.30428. 27787881 PMC5319890

[ref5] Chan E-Y, Glass G, Chua K-C, et al. (2018) Relationship between mastery and caregiving competence in protecting against burden, anxiety and depression among caregivers of frail older adults. *The Journal of Nutrition, Health and Aging* 22, 1238–1245. doi:10.1007/s12603-018-1098-1. PMC6302747 30498832

[ref6] Cox VC, Schepers VP, Ketelaar M, et al. (2018) A validation study of the Caregiver Mastery Scale for partners of patients with acquired brain injury. *Clinical Rehabilitation* 32, 493–500. doi:10.1177/0269215517732821. 28956478

[ref7] Cunningham RM, Walton MA and Carter PM (2018) The major causes of death in children and adolescents in the United States. *New England Journal of Medicine* 379, 2468–2475. doi:10.1056/NEJMsr1804754 30575483 PMC6637963

[ref8] De Schepper S, Vercauteren T, Tersago J, et al. (2016) Post-Traumatic Stress Disorder after childbirth and the influence of maternity team care during labour and birth: A cohort study. *Midwifery* 32, 87–92. doi:10.1016/j.midw.2015.08.010. 26410818

[ref9] Desjardins L, Solomon A, Shama W, et al. (2022) The impact of caregiver anxiety/depression symptoms and family functioning on child quality of life during pediatric cancer treatment: From diagnosis to 6 months. *Journal of Psychosocial Oncology* 40, 790–807. doi:10.1080/07347332.2021.2015646. 35016592

[ref10] Ding Y, Liu C, Xu H, et al. (2022) Effect of social support on illness perception in patients with atrial fibrillation during “Blanking Period”: Mediating role of sense of mastery. *Nursing Open* 10, 115–122. doi:10.1002/nop2.1284. 35855521 PMC9748061

[ref11] Dinkel A and Herschbach P (2018) Fear of progression in cancer patients and survivors. *Recent Results in Cancer Research* 210, 13–33. doi:10.1007/978-3-319-64310-6_2 28924677

[ref12] Fenton ATHR, Keating NL, Ornstein KA, et al. (2022) Comparing adult-child and spousal caregiver burden and potential contributors. *Cancer* 128, 2015–2024. doi:10.1002/cncr.34164. 35285946 PMC9038651

[ref13] Greer JA, Jacobs JM, El-Jawahri A, et al. (2018) Role of patient coping strategies in understanding the effects of early palliative care on quality of life and mood. *Journal of Clinical Oncology* 36, 53–60. doi:10.1200/JCO.2017.73.7221. 29140772 PMC5756320

[ref14] Gurtovenko K, Fladeboe KM, Galtieri LR, et al. (2021) Stress and psychological adjustment in caregivers of children with cancer. *Health Psychology* 40, 295–304. doi:10.1037/hea0001070. 34152783 PMC9053835

[ref15] Hinds PS, Gattuso JS, Fletcher A, et al. (2004) Quality of life as conveyed by pediatric patients with cancer. *Quality of Life Research* 13, 761–772. doi:10.1023/B:QURE.0000021697.43165.87. 15129886

[ref16] Hwang Y, McPhillips MV, Huang L, et al. (2023) Better caregiver mastery is associated with less anxiety in individuals with cognitive impairment. *BMC Nursing* 22, 307. doi:10.1186/s12912-023-01471-x. PMC10483800 37674161

[ref17] Kabia FM, El Fakiri F, Heus M, et al. (2022) Taking care of older caregivers who lose control: The association between mastery and psychopathology. *Archives of Gerontology and Geriatrics* 101, 104687. doi:10.1016/j.archger.2022.104687 35305440

[ref18] Kan K, Fierstein J, Boon K, et al. (2021) Parental quality of life and self-efficacy in pediatric asthma. *Journal of Asthma* 58, 742–749. doi:10.1080/02770903.2020.1731825. PMC7720799 32072838

[ref19] Lawton MP, Kleban MH, Moss M, et al. (1989) Measuring caregiving appraisal. *The Journals of Gerontology* 44, P61–71. doi:10.1093/geronj/44.3.p61 2715587

[ref20] Li Q, Lin Y, Hu C, et al. (2016) The Chinese version of hospital anxiety and depression scale: Psychometric properties in Chinese cancer patients and their family caregivers. *European Journal of Oncology Nursing* 25, 16–23. doi:10.1016/j.ejon.2016.09.004. 27865248

[ref21] Li Y, Wang K, Yin Y, et al. (2018) Relationships between family resilience, breast cancer survivors’ individual resilience, and caregiver burden: A cross-sectional study. *International Journal of Nursing Studies* 88, 79–84. doi:10.1016/j.ijnurstu.2018.08.011. 30212748

[ref22] Lim ZX, Chua WL, Lim WS, et al. (2022) Psychometrics of the Pearlin Mastery Scale among family caregivers of older adults who require assistance in activities of daily living. *International Journal of Environmental Research & Public Health* 19, 4639. doi:10.3390/ijerph19084639. PMC9027604 35457504

[ref23] Liu M, Tang W, Zhang Y, et al. (2023) Decisional conflict, caregiver mastery, and depression among Chinese parental caregivers of children with leukemia. *BMC Psychiatry* 23(1), 625. doi:10.1186/s12888-023-05084-1. PMC10463635 37641015

[ref24] Ljungman L, Cernvall M, Grönqvist H, et al. (2014) Long-term positive and negative psychological late effects for parents of childhood cancer survivors: A systematic review. *PLoS One* 9, e103340. doi:10.1371/journal.pone.0103340. PMC4110004 25058607

[ref25] Loh KP, Mohamed MR, Kadambi S, et al. (2021) Caregiver-oncologist prognostic concordance, caregiver mastery, and caregiver psychological health and quality of life. *The Oncologist* 26, 310–317. doi:10.1002/onco.13699. 33523583 PMC8018313

[ref26] Melaiu O, Lucarini V, Giovannoni R, et al. (2022) News on immune checkpoint inhibitors as immunotherapy strategies in adult and pediatric solid tumors. *Seminars in Cancer Biology* 79, 18–43. doi:10.1016/j.semcancer.2020.07.001. 32659257

[ref27] Mellon S, Kershaw TS, Northouse LL, et al. (2007) A family-based model to predict fear of recurrence for cancer survivors and their caregivers. *Psychooncology* 16, 214–223. doi:10.1002/pon.1074 16906624

[ref28] Montpetit MA and Tiberio SS (2016) Probing resilience: Daily environmental mastery, self-esteem, and stress appraisal. *The International Journal of Aging and Human Development* 83, 311–332. doi:10.1177/0091415016655162 27357303

[ref29] Nyagetuba JKM and Hansen EN (2017) Pediatric solid tumors in Africa: Different biology? *Current Opinion in Pediatrics* 29, 354–357. doi:10.1097/MOP.0000000000000483 28319559

[ref30] Sayal P, Rizakos S, Lam E, et al. (2023) A qualitative exploration of parental caregivers’ experience caring for children who have survived medulloblastoma. *Pediatric Blood and Cancer* 70(9), e30534. doi:10.1002/pbc.30534 37391864

[ref31] Scarpelli AC, Paiva SM, Pordeus IA, et al. (2008) Measurement properties of the Brazilian version of the Pediatric Quality of Life Inventory (PedsQL) cancer module scale. *Health and Quality of Life Outcomes* 6, 7. doi:10.1186/1477-7525-6-7. PMC2266904 18211688

[ref32] Schepper F, Abel K, Herschbach P, et al. (2015) Fear of progression in parents of children with cancer: Adaptation of the fear of progression questionnaire and correlates. *Klinische Pädiatrie* 227, 151–156. doi:10.1055/s-0035-1545352. 25811743

[ref33] Seyedreza M, Mahsa M, Parvaneh V, et al. (2021) The role of family caregiver’s sense of coherence and family adaptation determinants in predicting distress and caregiver burden in families of cancer patients. *Indian Journal of Palliative Care* 27(1), 47–53, doi:10.4103/IJPC.IJPC_112_20. 34035617 PMC8121216

[ref34] Shin H and Park C (2024) Mastery is central: An examination of complex interrelationships between physical health, stress and adaptive cognition, and social connection with depression and anxiety symptoms. *Frontiers in Psychiatry* 15, 1401142. doi:10.3389/fpsyt.2024.1401142 PMC11094708 38751422

[ref35] Steliarova-Foucher E, Stiller C, Lacour B, et al. (2005) International classification of childhood cancer, third edition. *Cancer* 103, 1457–1467. doi:10.1002/cncr.20910 15712273

[ref36] Stieb S, Fischbeck S, Wagner W, et al. (2018) High psychosocial burden in relatives of malignant brain tumor patients. *Clinical Neurology and Neurosurgery* 170, 1–6. doi:10.1016/j.clineuro.2018.04.023. 29709767

[ref37] Yang Y, Zhang Y, Liang L, et al. (2022) Fear of progression and its associated factors in parents of children undergoing cancer treatment: A cross-sectional study. *Psychooncology* 31, 1737–1744. doi:10.1002/pon.6027. 36073576

[ref38] Zarit SH, Todd PA and Zarit JM (1986) Subjective burden of husbands and wives as caregivers: A longitudinal study. *The Gerontologist* 26, 260–266. doi:10.1093/geront/26.3.260 3721233

